# Glycemic Responses, Glycemic Index, and Glycemic Load Values of Some Street Foods Prepared from Plantain (*Musa* spp., AAB Genome) in Côte d’Ivoire

**DOI:** 10.3390/foods6090083

**Published:** 2017-09-16

**Authors:** Camille Adam Kouamé, Nestor Kouakou Kouassi, Jacko Rhedoor Abodo, Kingsley Kwadwo Asare Pereko, Maria Cristina Casiraghi, Denis Yao N’dri, Georges N’guessan Amani

**Affiliations:** 1 Food Biochemistry and Tropical Products Technology Laboratory, Nutrition Section, Department of Food Science and Technology, University Nangui Abrogoua, P.O. Box 801 Abidjan 02, Cote D’Ivoire; nestorkksi@yahoo.fr; 2 Food Biochemistry and Tropical Products Technology Laboratory, Biochemistry and Food Technology Section, Department of Food Science and Technology, University Nangui Abrogoua, P.O. Box 801 Abidjan 02, Cote D’Ivoire; ndri_denis@yahoo.fr (D.Y.N.); amanigeorges@yahoo.fr (G.N.A.); 3 Endocrinology Diabetes Nutrition, CHU Yopougon, P.O. Box 632 Abidjan 23, Cote D’Ivoire; sfadabidjan@yahoo.fr; 4 Department of Community Medicine, School of Medical Sciences, University of Cape Coast, Cape Coast, Ghana; kpereko@gmail.com; 5 Department of Food, Environmental and Nutritional Sciences (DeFENS), Human Nutrition Unit, University of Milan, Via Celoria n. 2, 20133 Milan, Italy; maria.casiraghi@unimi.it

**Keywords:** diabetes mellitus, glycemic load, glycemic index, plantain cultivar, snack foods

## Abstract

The glycemic index (GI) and glycemic load (GL) of four culinary preferences including five local street dishes prepared from three varieties of plantain at different maturity stages was determined. The GI was obtained following ISO/FDI 26642:2010 protocol, and the GL was calculated from test foods’ GI, considering the amount of available carbohydrate in the traditional portion size. GI values were 44 for Klaclo (with Ameletiha variety at all black stage), 39 for Aloco (with Agnrin variety at full yellow stage), 39 for Aloco (with Agnrin variety at full yellow with black spots stage); 45 for Chips (with Ameletiha variety at green stage) and 89 for Banane braisée (with Afoto variety at light green stage). GI values were inversely correlated with the total sugar and carbohydrate in foods (*p* < 0.01), and no relationship existed between the GI values and the amount of protein (*p* = 0.89). Except for Chips (GL = 12), the GLs of the others foods were high (GL > 20). Contrary to Banane braisée, the consumption of Klaclo, Aloco, and Chips may promote the control of postprandial glucose response. Data provides the first GI published values of plantain-based foods commonly consumed in the urban area of Abidjan (Côte d’Ivoire).

## 1. Introduction

As a global commodity for food security, plantain (*Musa paradisiaca normalis*) has received attention in recent years by nutritionists, agronomists, and agriculturalists. Plantain has been recognized as an important food crop with high potential to improve food security globally considering its production, processing, and utilization [1], and there is a wide body of knowledge about the technology, chemistry, post-harvest physiology, and biochemistry of plantain [2,3,4]. Plantain is a staple food and an important source of carbohydrates for millions of people worldwide, particularly in sub-tropical countries, with global production estimated at 37.877 million 805 tons in 2013. It is the third food crop in Côte d’Ivoire, with an annual production of more than 1.624 million 354 tons [5] and an estimated consumption of 120 kg/capita/year [6]. In addition to its high domestic demand, plantain has a high market value both sub-regionally and internationally. Mature plantain pulp and derivative products have a high nutritional value in terms of micronutrients content including calcium, carotenoids, ascorbic acid, and zinc [7]; this composition varies according to the variety and the cultivar, as well as to the stage of fruit maturation. In particular, studies on post-harvest physiology show that plantain composition changes dramatically during ripening; plantain starch is progressively converted into sugars as ripening progresses, likely because of the increased activities of several endogenous enzymes [8]. The fruits of plantain are generally subject to post-harvest culinary processes that take into account the variety, the stage of maturity, and the addition of other ingredients [1]. Some culinary preparations such as roasted or fried plantain, plantain chips, boiled plantain, or pounded plantain, obtained from different varieties, represent a common staple food in many West African Countries—particularly Côte d’Ivoire, Ghana, Nigeria, and Togo [9]. Other preparations found in certain countries include plantain pastry lined with beans or with green leafy vegetables, plantain fritters, plantain pulp cooked with water, palm oil, goat or meat, salt and various spices, etc. [7]. In Côte d’Ivoire, plantains are consumed at all stages of ripeness. Recent data gathered from representative samples of consumers during surveys in urban areas of Abidjan (Côte d’Ivoire) showed four preferred culinary patterns of plantain consumption, according to variety and maturity of fruit, such as fried plantain called “Aloco”, fritters plantain called “Klaclo”, plantain chips “Chips” and roasted plantain “Banane braisée” [10]. Plantain chips are deep-fried thin slices (1.2–0.8 mm thick) of fruits. Fried plantains as for Aloco are thick slices in cubes of peeled ripe fruits, salted and fried in vegetable oil. Fritters plantain (Klaclo) is prepared with blended over-ripe fruit of plantain mixed with corn flour (30–50%), salt, and other spices, modelled into balls and fried in vegetable oil. Aloco and Klaclo are very popular dishes made in the small roadside restaurants and now in good restaurants called “allocodromme” in Côte d’Ivoire. This dish is usually served with grilled fish or hard-boiled egg and onion-tomato sauce and consumed in the afternoon as snack. It has other names such as Dodo in Benin and Amadan in Togo or Dodo-Ikere in Nigeria. Banane braisée (or “blissi”, as it is locally called) consist of the entire pulp of unripe or half-ripe plantain, roasted on heated charcoal with regular turning over to prevent the plantain from charring [11]. Women mainly carry out the preparation of chips, Aloco, fritters, and roasted plantain on the streets, an activity that often represents their principal source of income [10]. From a nutritional point of view, the wide diffusion of these plantain-based meals could have a significant impact on glucose metabolism, thereby contributing to the long-term development of metabolic disorders such as diabetes mellitus, which has become an issue of public health concern in Côte d’Ivoire [12]. Although there have been several reports on the nutritional properties of plantain [4], no data are available about the glycemic index (GI) or glycemic load (GL) of these street foods. This research was therefore undertaken to evaluate the GI and GL of several street foods, prepared with traditional recipes, from three local varieties of plantain at different stages of fruit ripeness.

## 2. Materials and Methods

### 2.1. Setting

The study was performed at the Department of Food Sciences and Technology of the University Nangui Abrogoua using internationally recognized GI methodology [13,14]. All clinical procedures were carried out at the Endocrinology and Diabetology Center, CHU Yopougon (Félix Houphouet Boigny University), Abidjan, Côte d’Ivoire. This study was conducted according to the principles of the Helsinki Declaration and approved by the Ethical Committee of the Félix Houphouet Boigny University Research, Cocody. Written informed consent was obtained from all subjects prior to participation. Participants were informed about the complete details of the study protocol and were given the opportunity to ask questions or to withdraw from the study at any time.

### 2.2. Materials

The reference food glucose (Glucose pur anhydre, COOPER, Place Lucien Anvert) was from Arts Pharmacy Limited (Abidjan-District, Côte d’Ivoire). Fresh and mature plantain fruit of three varieties—Afoto variety (*Musa* spp., AAB group, Queensland, Australia, cv False Horn), Agnrin variety (*Musa* spp., AAB group, cv French Horn), and Ameletiha variety (*Musa* spp., AAB group, Queensland, Australia, cv French)—were used in this experiment. Fruits were purchased in the local market of Yopougon-Siporex, Abidjan-District (Côte d’Ivoire) in quantities sufficient to conduct all tests. In order to make samples more homogeneous, each batch of plantain was bought on a single day from the same seller and stored in the laboratory under the same conditions, at room temperature (28 ± 2 °C) and relative humidity of 80–90%.

### 2.3. Subjects

The study involved a group of 30 (23 men and 7 women) healthy non-obese and physically active volunteers. They were recruited from the University Nangui Abrogoua among the staff and students through advertisement and were selected based on age (18–40 years), BMI (19–26 kg/m^2^), and fasting blood glucose value (4–5.5 mmol/L). Glycated hemoglobin (HbA1c) was also evaluated, and subjects with an HbA1c <8% were included in the study. Smokers were excluded from the study. Anthropometric measurements were carried out for all the subjects using standardized methods before the start of the study. Height was recorded to the nearest centimeter using a Stadiometer (Seca Limited, Birmingham, West Midlands, UK) with the subjects standing erect without shoes. Body weight was recorded using the Tanita BC-418 MA (Tanita UK Limited, Yiewsley, Middlesex, UK) with the subjects wearing light clothing and no shoes, and blood pressure was measured with an automatic device (A & D Company Ltd., Tokyo, Japan). Determination of HbA1c was assessed with a Bio-Rad DiaSTAT™ Hemoglobin A1C Analyzer (Bio-Rad Laboratories Inc., Hercules, CA, USA). Subjects’ characteristics are summarized in Table 1. During the study, subjects were advised to maintain their habitual daily activities without any change in their physical activities.

### 2.4. Test Foods—Collection of Samples and Preparation of Experimental Diets

Five local plantain-based dishes were prepared at the Food Biochemistry and Tropical Products Technology laboratory of University Nangui Abrogoua. These dishes were chosen on the basis of their frequency of consumption by the population of the urban areas of Abidjan [10]. They included fried plantain “Aloco” prepared from fruits at two stages of maturity—the full yellow stage (aag6) and the full yellow with black spots (aag7); fritters plantain “Klaclo” from fruit at the black stage of maturity (kam8); plantain chips from the green stage (Cam1); and charcoal-roasted “Banane braisée” from the light green stage of maturity (raf2). These stages correspond to preferential uses in traditional culinary preparations. Samples of the various food products were prepared by roasting and frying according to the traditional methods as described by Aboua et al. [11]. Table 2 lists the plantain species employed in the study, their ripening stages, and cooking/processing methods applied to formulate the test foods.

### 2.5. Proximate Analysis

All foods were tested immediately after cooking. Moisture, ash, lipids, and protein were assessed by following AACC International approved Methods n. 44-15.02, 08-01.01, 30-10.01, and 46-12.01, respectively. The total dietary fiber content was evaluated in accordance with the method of Prosky [15]; available carbohydrates (AvCHO) were calculated by difference as suggested by FAO/WHO procedure [13]. Total sugars (TS) were determined using the 3,5-dinitrosalicylic acid method [16]. All the reported determinations were carried out in triplicate. 

### 2.6. Glycemic Index Testing Procedures

The study was performed in accordance with the international standard GI testing protocol [14], in line with procedures recommended by the FAO/WHO Expert Consultation [13]. Subjects were invited to attend the test sessions on seven consecutive occasions with a 2-day interval between test days. Fifty (50) grams of anhydrous glucose powder dissolved in 250 mL water was used twice as the reference food; plantain dishes were prepared on the day of testing following the common practices used by the local food sellers in Côte d’Ivoire. All foods were tested in portions containing equivalent available carbohydrate amounts (50 g). On the day before a test, subjects were asked to restrict their intake of alcohol and caffeine-containing drinks and to avoid intense physical activity. The order of test foods was randomized, and all the foods were tested after a 12-h overnight fast. Blood glucose concentrations were measured in the capillary whole blood obtained by finger prick (Accu-Chek^®^ Fastclix Lancing Device, Castle Hill, NSW, Australia) in the fasted state and at 15, 30, 45, 60, 90, and 120 min after the start of the meal. Blood glucose was measured using a calibrated Accu-Chek^®^ Performa glucometer (Accu-Chek Performa, Roche Diagnostic, Castle Hill, NSW, Australia). 

### 2.7. Calculation of Glycemic Index and Glycemic Load

The incremental area under the post-prandial blood glucose curve (iAUC), ignoring the area beneath the baseline, was calculated geometrically for each tested food [17], and the GI was evaluated as a percentage of the mean iAUC of the reference glucose solution consumed by the same subject (GI = iAUC test food/iAUC reference food × 100). When the individual GI values for any subject fell outside the range of values calculated as mean ± 2 SD (standard deviation), this result was considered as an outlier and was thus excluded from the mean GI calculation. The glycemic load (GL) of a specific serving of each food was calculated using the formula: GL = GI food/100 × g of available carbohydrates in the portion. Based on the consumption habits observed in Côte d’Ivoire [10], the GL of the different tested foods was calculated considering the portions reported in Table 3.

### 2.8. Statistical Analysis

Data processing was carried out using SPSS software (version 17.0, SPSS, Chicago, IL, USA). Data are presented as means ± standard deviation (SD) or means ± standard error of mean (SEM), as indicated. Comparisons between the foods’ composition were made by using one-way analysis of variance (ANOVA) and Tukey’s multiple comparisons test. For GI data analysis, differences in postprandial blood glucose concentrations at any time points, iAUCs, GI, and GL values were evaluated using repeated-measures analysis of variance (RM-ANOVA). Spearman’s correlation coefficient was used to assess the relationship between GI values and macronutrient content of tested foods. Statistical significance was set at *p* < 0.05.

## 3. Results and Discussion

### 3.1. Chemical Composition of Meals

Energy and proximate composition of tested foods are shown in Table 3. The dry matter content of the meals varied from 44.5 g/100 g for Banane braisée to 66.4 g/100 g for Chips. As expected, plantain meals contain low amounts of proteins, varying in a range of values from 4.4 g/100 g dw (Aloco aag6) to 8.8 g/100 g dw (Aloco aag7), but a high lipid content. In particular, except for Banane braisée which showed a low lipid content (0.3 g/100 g dw), in Klaclo, Aloco (aag6 and aag7), and Chips, lipid levels were 14.1, 12.4, 11.6, and 10.9 g/100 g dw, respectively. Carbohydrates were the main nutrient present in meals, ranging from 78 to 93 g/100 g dw, with amounts of total sugar varying from 4 to 12.5 g/100 g dw. Total dietary fiber content was low and very similar among meals. These results are not surprising, since several previous studies have shown that the plantain is known for being very rich in starch and an excellent source of energy [18,19]. However, statistical analysis showed significant differences (*p* < 0.05) between the means of the variables estimated in the proximate composition of the test foods (Table 3). These differences could partly be attributed to certain inherent characteristics in each plantain variety, such as genotype, the conditions of cultures, and nature of soils [19], as well as the variability in the traditional processing/cooking methods used (Table 1). Numerous studies have reported that plantain is poor in fiber, lipid, and protein [18,19,20], and the nutritional composition of plantain is diversely affected by processing methods [11], as shown by our data (Table 2 and Table 3). Moreover, as expected, fried plantain contained the highest amount of lipid and dry matter compared to the roasted plantain (Table 3). Aloco aag6 and aag7, Chips, and fritter plantain (Klaclo) probably absorbed great amounts of oil, whereas the unripe plantain (roasted plantain) may have lost lipid during the roasting process, as explained in an earlier study [11]. Furthermore, these authors suggested that palm oil frying presents some nutritional advantages due to a vitamin and lipid enriching effect on the plantain meals, increasing its dry matter unlike the roasting process. These facts are important, because a high moisture content in food has been shown to accelerate microbial growth and food spoilage [21]. The serving sizes calculated to contain 50 g available carbohydrate are shown in Table 4. The GI testing portion differed considerably with the smallest portion calculated for Chips (93.8 g) and the largest portion for the Banane braisée (122.9 g).

### 3.2. Glycemic Responses, Glycemic Index, and Glycemic Load of Test Meals

The mean intra-individual CV of glycemic responses to the two 50 g glucose standard tests for the thirty subjects was about 4%, and the mean inter-individual CV in glycemic response to the standard tests was 11%. These values are consistent with reported data that low mean within-subject variation (reference CV < 30%) is required for accuracy [17]. 

**Figure 1:**
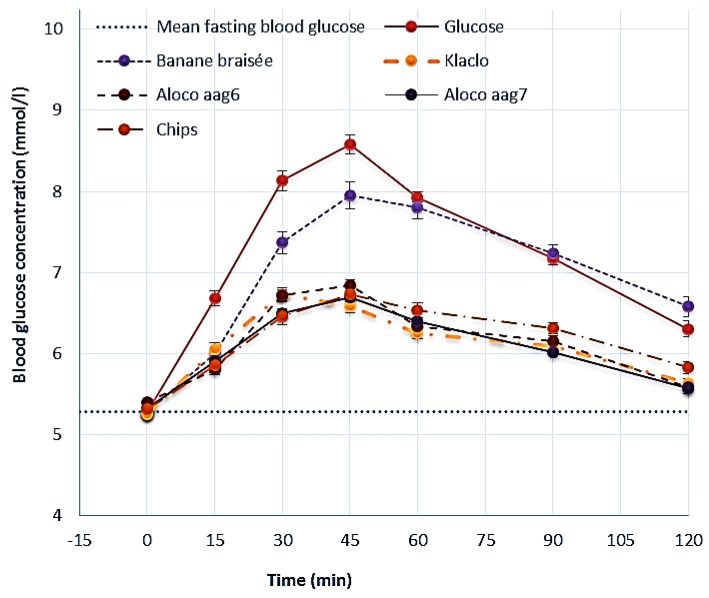
Glycemic response curves elicited by 50 g available carbohydrate portions of reference glucose, Banane braisée, Klaclo, Aloco aag6, Aloco aag7, and Chips. Values are the mean for 30 subjects with their SEM represented by vertical bars.

The mean post-prandial blood glucose curves of each test meal and reference food (glucose solution) are shown in Figure 1. Blood glucose concentration increased to the maximum value at 45 min for all the test foods and then decreased until 120 min. There were no significant variations between subjects regarding the mean basal blood glucose concentration assessed before ingestion of the test meals in all seven occasions. Blood glucose levels evaluated at each time point after the consumption of Banane braisée were not significantly different from those elicited by the glucose standard meal, except for times 30 and 45 min, in which capillary whole-blood glucose levels assessed for this meal were significantly lower than those observed for the glucose reference meal (*p* < 0.05). Furthermore, no significant differences were evidenced between blood glucose values at 15, 30, 45, 60, and 120 min after consumption of Klaclo, Aloco aag6, Aloco aag7, and Chips (*p* > 0.05). The postprandial glucose peak was consistently observed at 45 min for all the tested meals; the magnitude of the peak was significantly lower in subjects that received the fried plantain (*p* < 0.05) meals than in those who consumed roasted plantain (Banane braisée)—an effect that was also observable and significant for the iAUCs calculated for these meals (*p* < 0.001). The mean iAUC evaluated for Aloco (aag7), Aloco (aag6), Klaclo, and Chips were 94.5, 93.9, 107.5, and 110.1 mmol × min/L, respectively, resulting significantly (*p* < 0.05) lower than the iAUC calculated for Banane braisée.

The GI/GL values and classifications for each test meal are given in Table 4. The mean GIs assessed for plantain fried meals were low, ranging from 38 to 45; on the contrary, as expected from its iAUC value, Banane braisée showed the highest GI value of 89. In terms of variability, CVs evaluated for GI data were highest for Banane braisée (11%), followed by Aloco aag7 (7%), Aloco aag6 (4%), Klaclo, and Chips (3%). When RM-ANOVA with the post-hoc Tukey’s multiple comparison tests was applied to the all experimental data, significant (*p* < 0.05) differences were found among GI. In particular, Klaclo (GI = 44) and Chips (GI = 45) meals showed significantly (*p* < 0.05) higher values than those of evaluated for Aloco aag6 and Aloco aag7 (GI = 39). The higher GI of Banane braisée prepared at light green stage of fruit maturity assessed in this study is in contrast with that reported in other studies in which low and/or intermediate GI were observed [22]. This contrast could be related to the varietal differences of the species roasted. Nevertheless, in the case of Banane braisée, this food (the entire pulp of plantain) was processed on charcoal fire using dry heat. This may have resulted in the loss of water and the degradation of starches, thus increasing their digestibility. On the other hand, foods processed by frying in palm oil were found to have low GI (GI < 55) [23]. As reported in previous work [24,25], the lower GI observed upon frying compared to roasting could be attributed to the high lipid content, which promotes a slow rate of starch digestion as a consequence of the delay of gastric emptying. In fact, lipids are known to slow gastric emptying [26], as corroborated by the significant correlation (*p* < 0.05) assessed in this study between the GI values and the lipid content of meals. Lipids delay the transition time of the stomach contents to the duodenum, thus reducing starch hydrolysis rate and the absorption of monosaccharides through the microvilli of the epithelial cells of the jejunum as well as in upper part of the ileum, leading to positive effects on the postprandial glycemic and insulin responses [27]. However, even though Klaclo, Chips, and Aloco may result in lower GI values than Banane braisée, they are processed with increasing amounts of palm oil, and thus their consumption should not be promoted [26]. 

Although the dietary glycemic index is directly relevant in metabolic studies in which the total carbohydrate content is held constant, in free-living populations the amount of carbohydrate (e.g., as a percentage of energy) and its composition varies among individuals. Since the glucose and insulin responses depend on both the quantity and quality of the carbohydrate, the dietary glycemic load was used to represent both these traits of the carbohydrate intake. Indeed, high-GL diets increase the risk of diabetes by chronically increasing insulin demand, which in turn may lead to β-cell exhaustion, dysfunction, and apoptosis [28]. The GLs calculated for tested plantain foods appear high (GL > 20) [23], despite the fact that the majority of the meals (except for Banane braisée) had shown a low GI (GI < 55). The high GL values assessed can mainly be attributed to the large serving-size in which these plantain based meals are traditionally consumed. Considering the widespread consumption of these foods in Côte d’Ivoire, a suggestion to reduce the portion sizes of these street foods should therefore be recommended in the Ivoirian dietary guidelines, in an attempt to promote consumer behaviors which could favor a better control of glycemic metabolism. For optimal health, aim to keep a daily GL under 100 (www.glycemicindex.com). Thus, the portion sizes which are currently popular must be limited to 200 g for Banane braise, 300 g for Klaclo and Aloco, and 100 g for Chips. The relationships between meals composition and GI were investigated by bivariate correlations. As expected, correlation data analysis showed that the nutrient intake of the test meals influences their GI values. Lipid exhibited the strongest negative correlation with GI values (Spearman’s ρ = −0.542; *p* < 0.01), and no significant association was observed between the protein level of tested food and their GI values (Spearman’s ρ = 0.126; *p* > 0.01). This was expected, as previous findings from Henry et al. [29] indicated that a small amount of protein in foods—as observed in this study (4.4–8.8 g/100 g; Table 3)—does not significantly affect the glycemic response. At least 20–30 g dietary protein is needed to increase insulin responses sufficiently to reduce glycemic responses [30].

## 4. Conclusions

Our data set provides for the first time the GI values of some local plantain-based street foods commonly consumed in the urban area of Abidjan. Our results underline that meals such as Klaclo, Aloco, and particularly Chips may promote a low postprandial glucose response, in contrast with the opinion diffused among Ivorian population. However, despite their low GI, the GL assessed for this street food was quite high, suggesting a revision of portions commonly consumed. Moreover, these products are formulated with amounts of palm oil and thus should not be promoted. The importance and popularity of plantain in Côte d’Ivoire as a food crop dictate the need to characterize the glycemic impact (GI and GL) of all the foods prepared from plantain with the aim of better understanding their role in managing and/or preventing diseases related to glucose metabolism.

## Figures and Tables

**Table 1:** Baseline clinical and anthropometric characteristics (mean ± standard error of mean (SEM)) of subjects (*n* = 30) involved in the study.

Parameters	Sample		
	Mean	SEM	Range
Age (years)	30	0.5	25–35
Gender (male/female)	23/7	-	-
Body weight (kg)	63.3	1.3	47–74
Height (m)	1.7	0.0	1.6–1.9
BMI (kg/m^2^)	21.2	0.3	18.1–24.6
Fasting glucose (mmol/L)	4.6	0.1	4.1–5
HbA1c (%)	3.0	0.1	2.1–4.0
Systolic BP (mmHg)	107.7	1.7	90–120
Diastolic BP (mmHg)	73.0	1.3	60–90

BMI = body mass index, HbA1c = hemoglobin A1c, BP = Blood pressure.

**Table 2:** Composition and local cooking/processing methods applied to formulate the tested foods.

Food Tests	Code	Plantain Species (*Musa* spp., AAB Genome)	Ripening Stage at Use	Local Name	Cooking Method	Major Ingredients
Charcoal-roasted plantain	raf2	Afoto	light green (stage 2)	Banane braisée	Roasting	-
Fried plantain	aag6	Agnrin	full yellow (stage 6)	Aloco	Deep frying	Salt, refined palm oil
Fried plantain	aag7	Agnrin	full yellow with black spots (stage 7)	Aloco	Deep frying	Salt, refined palm oil
Fritters plantain	kam8	Ameletiha	all black (stage 8)	Klaclo	Deep frying	Salt, refined palm oil, corn flour (30%)
Chips plantain	cam1	Ameletiha	green (stage 1)	Chips	Deep frying	Salt, refined palm oil

**Table 3:** Proximate composition of tested meals.

Food Samples (Code)	Banane Braisée (raf2)	Klaclo (kam8)	Aloco (aag7)	Aloco (aag6)	Chips (cam1)
Dry matter (g/100 g)	44.5 ± 0.1 ^d^	63.7 ± 0.1 ^b^	66.2 ± 0.0 ^c^	63.6 ± 0.0 ^b^	66.4 ± 0.1 ^a^
Ash (g/100 g)	1.4 ± 0.0 ^c^	1.1 ± 0.0 ^d^	1.0 ± 0.0 ^e^	1.5 ± 0.0 ^b^	2.0 ± 0.0 ^a^
Proteins (g/100 g)	5.3 ± 0.0 ^c^	6.1 ± 0.0 ^b^	8.8 ± 0.0 ^a^	4.4 ± 0.0 ^d^	5.3 ± 0.0 ^c^
Lipids (g/100 g)	0.3 ± 0.1 ^e^	14.1 ± 0.2 ^a^	12.4 ± 0.0 ^b^	11.6 ± 0.1 ^c^	10.9 ± 0.0 ^d^
Total CHO (g/100 g) *	93.1 ± 0.1 ^a^	78.7 ± 0.2 ^d^	78 ± 0.0 ^e^	82.6 ± 0.1 ^b^	81.9 ± 0.1 ^c^
Total sugars (g/100 g)	6.5 ± 0.1 ^c^	12.5 ± 0.3 ^a^	10.3 ± 0.3 ^b^	9.8 ± 0.1 ^b^	4.0 ± 0.3 ^d^
Starch (g/100 g)	77.9 ± 0.2 ^a^	59.6 ± 0.0 ^e^	60.9 ± 0.2 ^d^	65.5 ± 0.2 ^c^	70.1±0.3 ^b^
Total dietary fiber (g/100 g)	1.7 ± 0.0 ^a^	1.6 ± 0.0 ^b^	1.6 ± 0.0 ^ba^	1.6 ± 0.0 ^ba^	1.7 ± 0.0 ^a^
Energetic Value (kcal/100 g) **	395.8 ± 0.4 ^e^	465.7 ± 1 ^a^	458.0 ± 0.3 ^b^	451.9 ±0.4 ^c^	446.7 ± 0.2 ^d^

Data are expressed on dry matter basis. Data in the same line with different superscript letter are significantly different (*p* < 0.05) as assessed by Tukey’s test. Data are means (standard deviation) of three independent experiments. * Calculated by difference of moisture content, ash, fiber, lipids and protein. ** Energetic value = 4 × %total carbohydrates + 4 × %protein + 9 × %lipids (kcal/100g)

**Table 4:** Glycemic index and glycemic load values of tested product.

Food	Code	Available CHO (g/100 g of Food Wet Weight)	Experimental Portion (g)	*n*	GI (Mean ± SEM)	Category ^1^	Serving Size	GL	Category ^2^
Banane braisée	raf2	41	122.9	28	88 ± 1.8 ^a^	High	178.8	64	High
Klaclo	kam8	49	101.8	28	44 ± 0.3 ^c^	Low	227.0	46	High
Aloco	aag6	50	99.0	29	39 ± 0.3 ^b^	Low	281.1	55	High
Aloco	aag7	52	97.0	29	39 ± 0.5 ^b^	Low	281.1	55	High
Chips	cam1	53	93.8	28	45 ± 0.3 ^c^	Low	49.4	12	Medium

^a,b,c^ Data in the same column with different letter superscripts are significantly different (*p* < 0.05); *n* = number of values included after outliers analysis. GI = Glycemic Index; GL = Glycemic Load; CHO = Carbohydrate. ^1^ Glycemic indexes were classified as high (≥70), medium (56–69), and low (≤55); ^2^ Glycemic loads were classified as high (≥20), medium (11–19), and low (≤10) (www.glycemicindex.com).
